# Development and Validation of Novel Biomarkers Related to M2 Macrophages Infiltration by Weighted Gene Co-Expression Network Analysis in Prostate Cancer

**DOI:** 10.3389/fonc.2021.634075

**Published:** 2021-06-29

**Authors:** Ning Xu, Ru-Nan Dong, Ting-Ting Lin, Tian Lin, Yun-Zhi Lin, Shao-Hao Chen, Jun-Ming Zhu, Zhi-Bin Ke, Fei Huang, Ye-Hui Chen, Xue-Yi Xue

**Affiliations:** ^1^ Department of Urology, Urology Research Institute, The First Affiliated Hospital of Fujian Medical University, Fuzhou, China; ^2^ Fujian Key Laboratory of Precision Medicine for Cancer, The First Affiliated Hospital, Fujian Medical University, Fuzhou, China; ^3^ Central Lab, Key Laboratory of Radiation Biology of Fujian Higher Education Institutions, The First Affiliated Hospital, Fujian Medical University, Fuzhou, China

**Keywords:** M2-TAMs, prostate cancer, CIBERSORT algorithm, weighted gene co-expression network analysis, biomarkers

## Abstract

M2-tumor-associated macrophages (TAMs) work as a promoter in the processes of bone metastases, chemotherapy resistance, and castration resistance in prostate cancer (PCa), but how M2-TAMs affect PCa has not been fully understood. In this study, we analyzed the proportion of tumor-infiltrating immune cells using the CIBERSORT algorithm, based on samples from the Cancer Genome Atlas database. Then we performed weighted gene co-expression network analysis to examine the modules concerning infiltrated M2-TAMs. Gene Ontology analysis and pathway enrichment analysis were performed for functional annotation and a protein–protein interaction network was constructed. The International Cancer Genomics Consortium cohort was used as a validation cohort. The red module showed the most correlation with M2-TAMs in PCa. Biological processes and pathways were mainly associated with the immune-related processes, as revealed by functional annotation. Four hub genes were screened: ACSL1, DLGAP5, KIF23 and NCAPG. Further validation showed that the four hub genes had a higher expression level in tumor tissues than that in normal tissues, and they were good prognosis biomarkers for PCa. In conclusion, these findings contribute to understanding the underlying molecular mechanisms of how M2-TAMs affect PCa, and looking for the potential biomarkers and therapeutic targets for PCa patients.

## Introduction

Prostate cancer (PCa) is a heterogeneous disease, ranging from an asymptomatic stage to an distantly metastatic or castration-resistant stage (1, 2). Guideline recommends radical prostatectomy or radical radiotherapy as the primary treatment for most patients with clinical localized PCa, and these patients usually have a good prognosis (3). As for patients with metastatic or castration-resistant disease, however, local treatments have limited efficacy for them. They eventually die when systemic treatment fails to control the progression of the disease (4). Although the number of patients with advanced disease has been substantially reduced and various novel therapeutic agents have presented notable success for patients with metastatic and castration-resistant PCa in recent years, some of them still experience disease progression, which may be attributed to individual differences at the genetic level (5–7). Therefore, it is urgent to elucidate the molecular mechanism of the disease and look for appropriate biomarkers, which will be beneficial to the diagnosis, treatment and prognosis prediction for PCa.

In recent years, it has been recognized that the tumor microenvironment (TME) is intimately involved in tumor development and progression (8–10). Tumor-associated macrophages (TAMs) are the main immune cells in TME. Generally, the macrophages are divided into two subsets depending on different polarized status: the classically activated (M1) and the alternatively activated (M2) macrophages (11). In inflammatory condition, classically activated M1-TAMs phenotype prevails in sites of inflammation; however, tumor development gradually promotes a phenotypic switch in which TAMs represent a immunosuppressive M2-TAMs phenotype (12). The presence of M2-TAMs has been related to poor clinical prognosis in several malignant diseases and is thought to affect disease outcome by stimulating angiogenesis, promoting distant metastasis, suppressing antitumor immunity and possibly by reducing the effectiveness of certain treatments (9). It was reported that M2-TAMs worked as a promoter in the processes of bone metastases, chemotherapy resistance, and castration resistance in PCa (13–15). However, the specific interactive mechanism between M2-TAMs and PCa has not been fully clarified. It is crucial to explored the key genes and molecules in this interaction process.

Weighted gene co-expression network analysis (WGCNA) is an algorithm that construct free-scale gene co-expression networks to explore the relationships between gene sets and clinical features (16). It has been widely used to screen the hub genes associated with clinical feature in different cancer types (17). In present study, we performed WGCNA to explore the role of TMAs and screen the potential biomarkers based on PCa gene expression data. We calculated the M2-TAMs proportion in these samples by CIBERSORT algorithm and then identified important modules and hub genes associated with the proportion of infiltrated M2-TAMs. This is the first utilization of WGCNA to identify M2-TAMs-related hub genes and biomarkers of PCa.

## Materials and Methods

### Data Collection and Preprocessing

**Figure 1:**
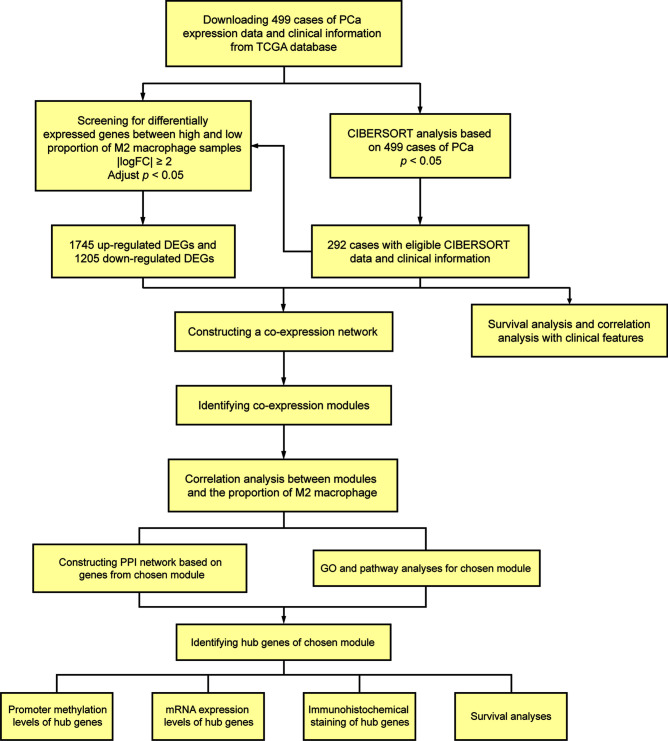
Flowchart detailing the study design and samples.

A total of 499 cases of PCa samples from the Cancer Genome Atlas (TCGA) database (https://portal.gdc.cancer.gov/), all of their expression profiles were downloaded. Cases were excluded due to inadequate clinical data. CIBERSORT algorithm was used to evaluate the cellular composition of immune cells. Meanwhile, clinical significance of each type of immune cells was evaluated using survival analyses and correlation analyses with clinical features. Thereafter, we screened differently expressed genes (DEGs) of samples with high and low ratio of M2-TAMs.WGCNA was performed to determine the module associated with M2-TAMs. Gene Ontology (GO) analysis and pathway enrichment analysis were performed for functional annotation of selected modules. A protein–protein interaction (PPI) network was built and hub genes were screened according to the degree of connectivity. Then, online databases were used for further validation. Besides, the additional independent cohort including 494 PCa samples from International Cancer Genomics Consortium (ICGC) database (https://dcc.icgc.org/) and cohort from TCGA database were used for survival analyses. **Figure 1** shows the flowchart detailing the study design and samples.

### Evaluation of Tumor-Infiltrating Immune Cells

CIBERSORT is a deconvolution algorithm that uses a set of reference gene expression values (a “signature matrix” of 547 genes) considered a minimal representation for each cell type and, based on those values, infers cell type proportions in data from bulk tumor samples of mixed cell types using support vector regression (18). Normalized gene expression data were used to infer the relative proportions of 22 types of infiltrating immune cells using the CIBERSORT algorithm. Briefly, gene expression datasets were prepared using standard annotation files and data uploaded to the CIBERSORT web portal (http://cibersort.stanford.edu/), with the algorithm run using the default signature matrix at 1,000 permutations. CIBERSORT derives a *P*-value for the deconvolution of each sample using Monte Carlo sampling, providing a measure of confidence in the results. Finally, we selected 292 samples with a CIBERSORT P-value of < 0.05, from the analyzed samples of 499 PCa cases.

### Differentially Expressed Genes Screening

The DEGs between samples with high and low proportions of M2-TAMs in TCGA cohort were analyzed using the “limma” R package. As described previously, adjusted P-value <0.05 and |logFC| ≥2 was set as the cut-off criterion for better accuracy and significance (19, 20).

### Construction of Co-Expression Network

Data quality assessment and pre-processing were performed at first. Then, WGCNA algorithm was used to construct a scale-free co-expression network for the DEGs. After that, a weighted adjacency matrix was established, as calculated by a_mn_ = |c_mn_|^β^ (Where c_mn_ is Pearson’s correlation between gene m and gene n, and where a_mn_ is adjacency between gene m and gene n). Considered as a soft-thresholding parameter, β can emphasize strong relations between genes while penalize weak correlations. We chose a proper power of β according to the mean connectivity. We transformed the adjacency into a topological overlap matrix (TOM), which is used to describe corresponding dissimilarity (1-TOM) (21). Subsequently, according to the TOM-based dissimilarity with a minimum size (gene group) of 30 for the genes dendrogram, we constructed average linkage hierarchical clustering aimed to classify genes that have similar expression profile into modules.

### Identification of Modules Related to the Proportion of M2 Macrophage Cells

Two methods were used for identification of modules associated with clinical features of PCa. Gene significance (GS) defined as log10 transformation of the P-value (GS = lgP) in the linear regression between gene expression and the clinical characteristic and module significance (MS) considered as the average GS of all genes in the module were calculated, respectively. The module with the MS ranked first in all of the selected modules was regarded as the one with clinical significance. Module eigengene (ME) was defined as the first principle component in each gene modules to summarize the expression patterns of all genes into a single characteristic expression profile in a given module. The relation between each ME and clinical characteristic for identification of the relevant module was computed. Ultimately, we chose the module highly correlated to specific clinical features for further analysis.

### Functional Enrichment Analysis

Metascape (http://metascape.org/) and Webgestalt (http://www.webgestalt.org/) are online databases providing a comprehensive set of functional annotation tools for researchers to better understand biological meaning behind large list of genes (22). We uploaded genes to perform GO analysis and pathway enrichment analysis. P-value <0.05 was thought statistically significant.

### PPI Network and Hub Genes Selection

Search Tool for the Retrieval of Interacting Genes (STRING) is a biological database for constructing PPI networks, providing a system-wide view of interactions between each member (23). Genes of selected module were mapped to STRING to explore their relationships with each other. A combined score of >0.4 was set as the cut-off criterion as described previously (24). Then, we established PPI network using Cytoscape software (24). The genes with high degree of connectivity were selected as the hub genes.

### Validation of the Hub Genes by Online Database

UALCAN (http://ualcan.path.uab.edu/) is a portal for facilitating tumor subgroup gene expression and survival analyses (25). Expression levels in mRNA and promoter methylation levels of hub genes were revealed using UALCAN. Afterwards, we used the Human Protein Atlas (http://www.proteinatlas.org) for validation in immunohistochemistry aspect.

### Evaluation the Value of Hub Genes as Prognosis Biomarkers

Cohort containing 494 cases of PCa samples from ICGC database and Cohort containing 292 cases from TCGA database were used for survival analyses by Kaplan–Meier method. Patients were divided into high expression group and low expression group according to a cut-off value of mean expression of the hub genes. Afterwards, survival analyses for hub genes were performed. The hazard ratio (HR) with 95% confidence intervals and log-rank P-value was calculated and displayed.

### Statistical Analysis

For statistical analysis, we used GraphPad Prism 5.0 (GraphPad Software, San Diego, CA, USA) and SPSS version 22.0 software (SPSS, Chicago, IL, USA). The relationship between the proportion of M2-TAMs and clinicopathological parameters were analyzed using chi-square test. Survival curves plotted by the Kaplan–Meier method were compared to the log-rank test. P < 0.05 was considered statistically significant.

## Results

### Tumor-Infiltrating M2 Macrophage in PCa

**Figure 2:**
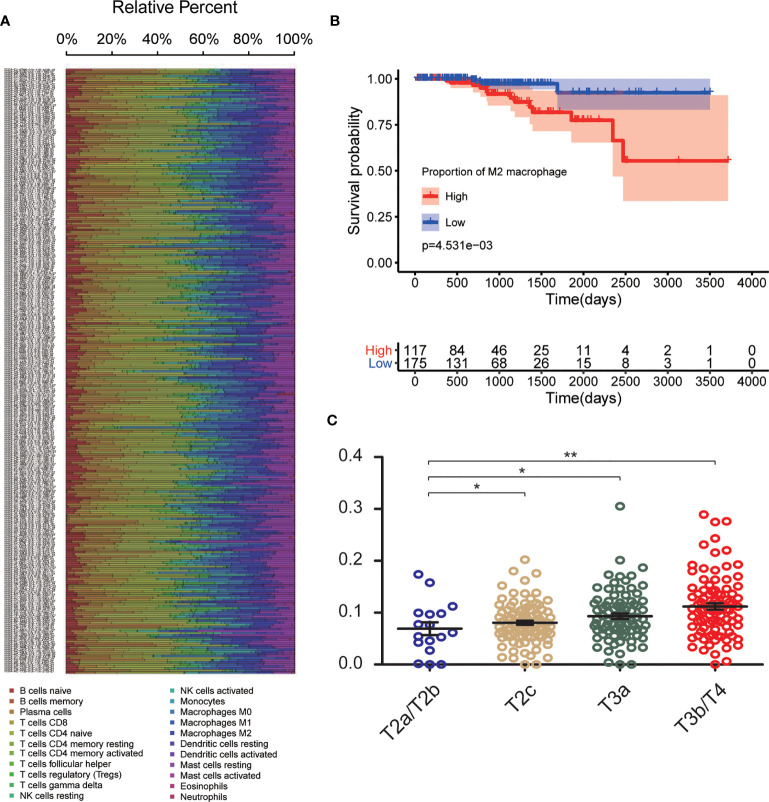
CIBERSORT analysis and clinical significance of M2-TAMs in PCa. **(A)** Relative percent of each type of immune cell in 292 PCa samples from TCGA cohort; **(B)** Proportion of M2-TAMs in different stages; **(C)** Overall survival between patients with high and low proportion of infiltrated M2-TAMs. *P < 0.05; **P < 0.01.

The CIBERSORT algorithm was used to investigate the 22 infiltrating immune cells subsets based on 292 PCa samples, whose clinical characteristics were shown in **Figure 2A**. Age, T stage, N stage, and survival status of the cases were collected. The results revealed that a higher proportion of infiltrating M2-TAMs was related to a more advanced stage (**Figure 2C**). Compared to patients with a low proportion of M2-TAMs, those with a high proportion of M2-TAMs had a worse prognosis (**Figure 2B**).

### DEGs Screening

Using the CIBERSORT algorithm, we detected the proportion of infiltrating M2-TAMs and the mRNA expression data from 292 PCa samples whose clinicopathology characteristics were summarized in **Table 1**. Using the average value of the proportion of M2-TAMs as a cut-off value, cases were divided into two groups, which one was 117 cases with a high proportion of M2-TAMs, another one was 175 cases with a low proportion of M2-TAMs. A total of 2,950 DEGs (1,745 upregulated and 1,205 downregulated) were chosen for subsequent analysis under the threshold of adjusted *P*-value <0.05 and |logFC| ≥2.

**Table 1:** Clinicopathological characteristics of 292 patients with PCa from TCGA cohort.

Variables	N	Fraction of M2 macrophage		*P*-value
		Low	High	
Total, n (%)	292	175	117	
Age				0.052
<60 y	118	79	39	
≥60 y	174	96	78	
T stage				<0.001*
T2	110	82	28	
T3	171	87	84	
T4	11	6	5	
N stage				0.394
N0	215	132	83	
N1	77	43	34	
Survival				0.006*
Yes	279	172	107	
No	13	3	10	

*P ＜ 0.05.

### Construction of Weighted Co-Expression Network and Identification of Key Module

**Figure 3:**
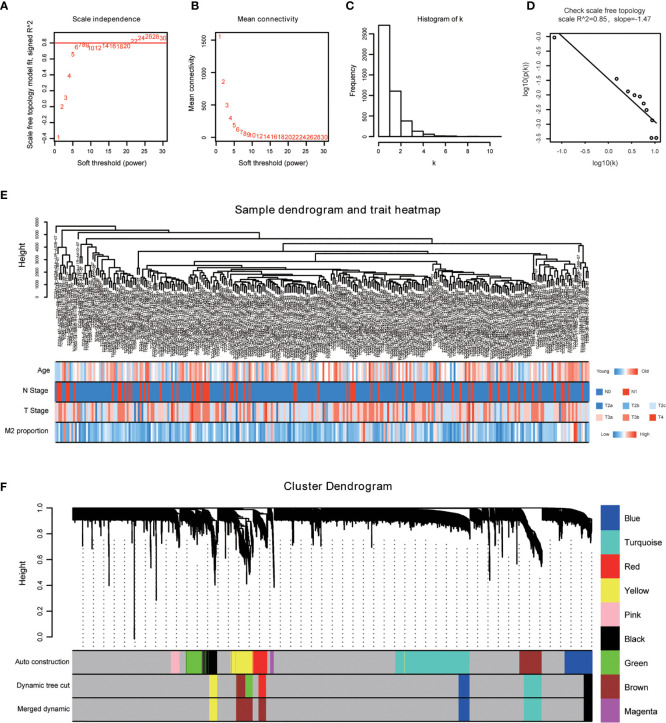
WGCNA was performed based on PCa samples from TCGA database. **(A)** Analysis of the scale-free fit index for various soft-thresholding powers; **(B)** Analysis of the mean connectivity for various soft-thresholding powers; **(C)** Histogram of connectivity distribution when β = 22; **(D)** Checking the scale free topology when β = 22; **(E)** The sample dendrogram and corresponding clinical characteristics; red: high, blue: low. **(F)** Cluster dendrogram of 292 samples with eligible data.

**Figure 4:**
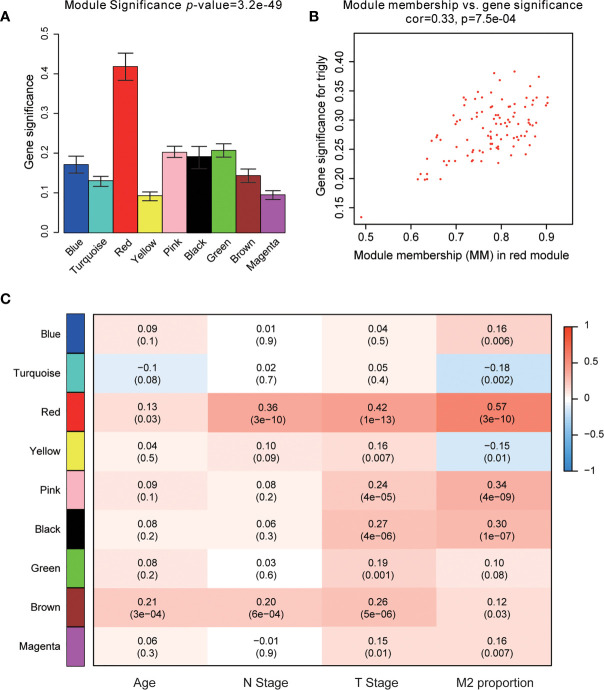
Identification of modules associated with clinical characteristics. **(A)** Distribution of average gene significance and errors in the modules associated with the proportion of M2-TAMs in PCa; **(B)** Scatter plot of module eigengenes in red module; **(C)** Heatmap of the correlation between module eigengenes and different clinical characteristics of PCa.

The power of β = 22 (scale free R^2^ = 0.85) was selected as the best soft-thresholding parameter (**Figures 3A, B** **)**. As can be seen from **Figures 3C, D**, the rationality test had a positive result. Then, the dendrogram of sample constructed shows the similarity among the samples, in which the clinical characteristics of each sample are displayed (**Figure 3E**). After that, nine modules were identified (**Figure 3F**). Two methods were used to test the relationship between each module and the M2-TAMs. Modules with a higher MS value were considered to have more connection with high infiltration of M2-TAMs, and we found that the MS of the red module was higher than those of any other modules (**Figure 4A**). Afterwards, the ME of the red module revealed a higher correlation with M2-TAMs than other modules (**Figure 4C**). Eventually, we identified the red module as the module most associated with high infiltration of M2-TAMs in PCa (**Figure 4B**).

### Functional Enrichment Analysis

**Figure 5:**
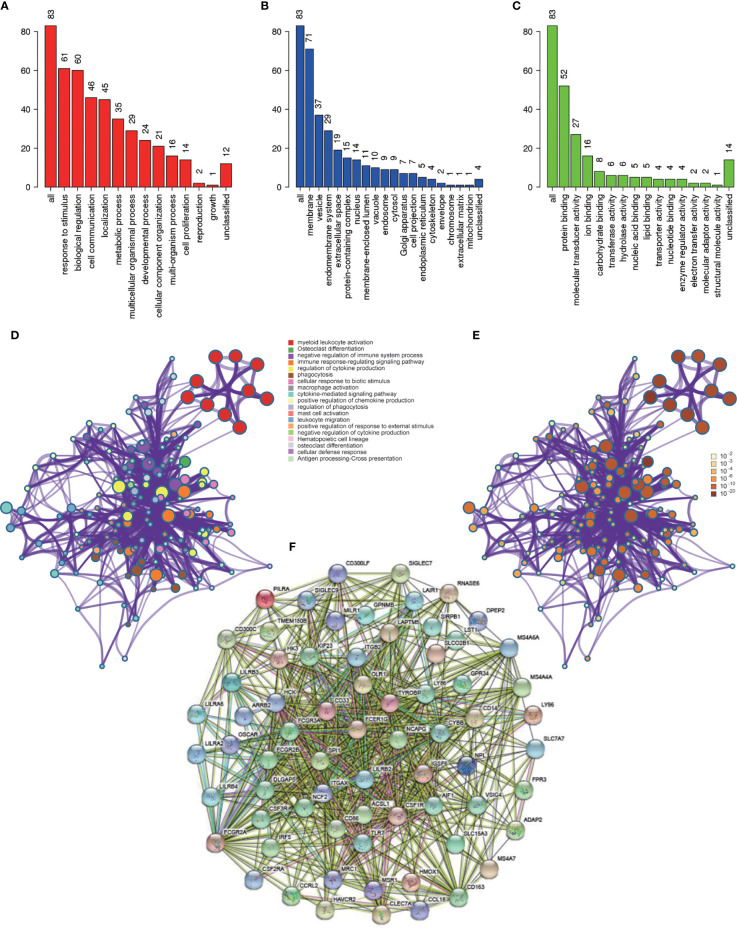
Functional enrichment analysis and construction of PPI network. **(A)** GO analysis of biological process for genes in red module; **(B)** GO analysis of cellular component for genes in red module; **(C)** GO analysis of molecular function for genes in red module; **(D)** Functional enrichment analysis for genes in red module. **(E)** *P*-value of each gene in the network; **(F)** PPI network constructed using STRING.

Functional enrichment analysis was conducted to look for the biological processes and pathways associated with red module. We used the tool of Webgestalt to perform GO analysis. GO analysis of biological process showed that genes in red module were mainly involved in response to stimulus, biological regulation, cell communication, localization and metabolic process (**Figure 5A**). GO analysis of cellular component showed that these genes were mainly enriched in membrane, vesicle, endomembrance system, extracellular space and protein-containing complex (**Figure 5B**). GO analysis of molecular function demonstrated that these genes were mainly involved in protein binding, molecular transducer activity, ion binding, carbohydrate binding and transferase activity (**Figure 5C**). Then we used Metascape to further investigate the relevant biological processes and pathways. The result revealed that the biological processes and pathways were mainly associated with the immune processes, such as myeloid leukocyte activation, osteoclast differentiation, negative regulation of immune system process, immune response-regulating signaling pathway and regulation of cytokine production (**Figures 5D, E** and **Table 2**).

**Table 2:** Functional enrichment analysis for genes in red module.

Category	Term	Count	%	*P*-value	*q*-value
GO Biological Processes	myeloid leukocyte activation	28	33.73	1.42E−07	2.82E−06
KEGG Pathway	Osteoclast differentiation	16	19.28	8.67E−07	1.24E−05
GO Biological Processes	negative regulation of immune system process	19	22.89	3.33E−05	3.15E−04
GO Biological Processes	immune response-regulating signaling pathway	21	25.30	5.94E−05	5.36E−04
GO Biological Processes	regulation of cytokine production	21	25.30	8.47E−05	7.20E−04
GO Biological Processes	phagocytosis	16	19.28	1.34E−04	1.07E−03
GO Biological Processes	cellular response to biotic stimulus	11	13.25	1.62E−03	1.06E−02
GO Biological Processes	macrophage activation	8	9.64	2.12E−03	1.35E−02
GO Biological Processes	cytokine-mediated signaling pathway	17	20.48	2.20E−03	1.40E−02
GO Biological Processes	positive regulation of chemokine production	6	7.23	6.27E−03	3.48E−02
GO Biological Processes	regulation of phagocytosis	7	8.43	6.36E−03	3.51E−02
GO Biological Processes	mast cell activation	6	7.23	7.51E−03	4.08E−02
GO Biological Processes	leukocyte migration	12	14.46	8.68E−03	4.59E−02
GO Biological Processes	positive regulation of response to external stimulus	10	12.05	9.05E−03	4.71E−02
GO Biological Processes	negative regulation of cytokine production	9	10.84	1.50E−02	7.44E−02
KEGG Pathway	Hematopoietic cell lineage	6	7.23	1.59E−02	7.82E−02
GO Biological Processes	osteoclast differentiation	6	7.23	1.59E−02	7.82E−02
GO Biological Processes	cellular defense response	5	6.02	1.59E−02	7.83E−02
Reactome Gene Sets	Antigen processing-cross presentation	6	7.23	1.65E−02	8.06E−02

### Construction of PPI Network and Identification of Hub Genes

PPI network was constructed with STRING (**Figure 5F**), and finally four hub genes were screened for further investigation. They were ACSL1 (Acyl-CoA Synthetase Long Chain Family Member 1), DLGAP5 (DLG Associated Protein 5), KIF23 (Kinesin Family Member 23) and NCAPG (Non-SMC Condensin I Complex Subunit G). The four genes were all of significantly clinical significance. The module membership values of them were 0.91, 0.82, 0.74 and 0.87, respectively, which were significant statistically. **Figure 4B** showed the scatter plot of module eigengenes in red module.

### Validation and Efficacy Evaluation of Hub Genes

**Figure 6:**
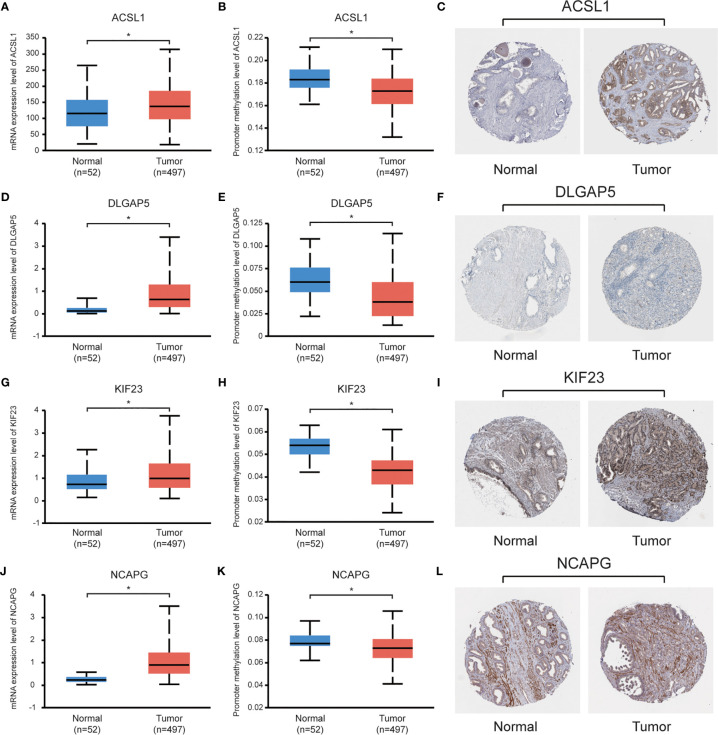
Validation of the hub genes using online databases. **(A**, **D**, **G**, **J)** Expression levels of the hub genes in PCa and normal prostate tissues; **(B**, **E**, **H**, **K)** Promoter methylation levels of the hub genes in PCa and normal prostate tissues; **(C**, **F**, **I**, **L)** Immunohistochemical staining of the hub genes in PCa and normal prostate tissues. *P < 0.05.

Data set from the online database UALCAN was used for validation. All four hub genes revealed higher expression levels in PCa samples, compared with normal tissues (**Figures 6A, D, G, J**). Besides, these hub genes revealed lower levels of promoter methylation in PCa tissues, when compared with the normal tissues (**Figures 6B, E, H, K**). Immunohistochemistry staining from the Human Protein Atlas database demonstrated that protein levels of the hub genes were significantly higher in PCa tissues than those in normal tissues (**Figures 6C, F, I, L**).

**Figure 7:**
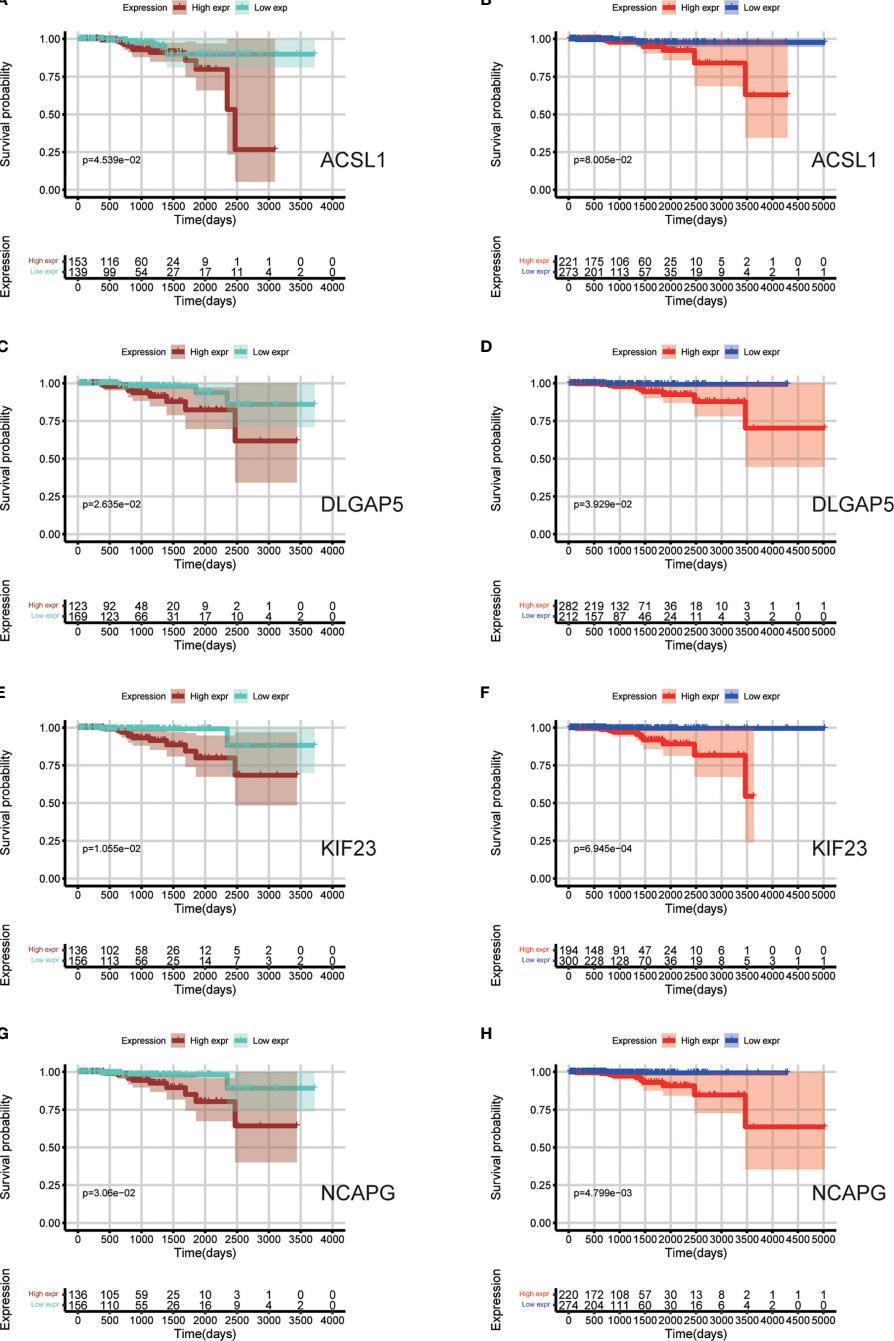
Survival analyses based on TCGA and ICGC cohorts to evaluate value of hub genes as prognosis biomarkers. **(A**, **C**, **E**, **G)** Overall survival between patients with high and low expression of the four hub genes based on TCGA cohorts; **(B**, **D**, **F**, **H)** Overall survival between patients with high and low expression of the four hub genes based on ICGC cohorts.

Data sets from the TCGA database and ICGC database (**Table 3**) were used for survival analyses to evaluate value of hub genes as prognosis biomarkers. It revealed that high expression levels of ACSL1 (HR 0.245 [0.092–0.427], *P* = 0.045) were related to poor overall survival in PCa patients, as well as DLGAP5 (HR 0.279 [0.108–0.494], *P* = 0.026), KIF23 (HR 0.305 [0.103–0.621], *P* = 0.011), and NCAPG (HR 0.271 [0.102–0.485], *P* = 0.031), based on TCGA cohort (**Figures 7A, C, E, G**). In addition, survival analyses based on ICGC cohort show consistent results: high expression levels of ACSL1 (HR 0.248 [0.101–0.418], *P* = 0.080), DLGAP5 (HR 0.416 [0.175–0.768], *P* = 0.039), KIF23 (HR 0.428 [0.117–0.696], *P <*0.001), and NCAPG (HR 0.231 [0.082–0.494], *P* = 0.005) were related to poor overall survival of PCa (**Figures 7B, D, F, H**).

**Table 3:** Clinicopathological characteristics of 494 patients with PCa from ICGC cohort.

Clinicopathological characteristics	Value
Age, y	
Mean ± SD	61.13 ± 6.80
Range	42–78
Race, n (%)	
White	295 (59.7)
black	14 (2.8)
Yellow	185 (37.5)
T stage, n (%)	
T2	251 (50.8)
T3	211 (42.7)
T4	32 (6.5)
N stage, n (%)	
N0	304 (61.5)
N1	127 (25.7)
unknown	63 (12.8)
Survival, n (%)	
Yes	483 (97.8)
No	11 (2.2)

## Discussion

Tumor cells and the microenvironment of the local host tissue interact with each other and form the TME. The TME plays a crucial role in tumor differentiation, tumor epigenetics, infiltration metastasis, and immune escape (26). Most therapies kill the tumor cells through cytocidal or cytostatic activity. In recent years, besides targeting tumor cells, therapies targeting the TME have been explored. For example, it has been demonstrated that targeting the vasculature can reduce tumor-related immunosuppression and thus improve therapeutic effects (27). Sipuleucel-T, an antigen-specific active immunotherapy agent, can sensitize the patient’s immune system against specific antigens or molecules, to train the system to attack the tumor, and has been proved to be beneficial for patients with metastatic castration-resistant prostate cancer (mCRPC) (28). As a major immune component in TME, TAMs have a tumor-suppressing polarized status called M1 subtype and a tumor-promoting polarized status called M2 subtype. The presence of M2-TAMs was related to poor clinical outcome in many cancers, such as squamous cell carcinoma, breast cancer and renal cell carcinoma (29–31). They cannot only promote the progression of the tumors, but also affect the efficacy of anti-tumor therapy (32, 33). In PCa, patients with higher proportions of M2-TAMs in TME had a higher death rate and a shorter time to biochemical recurrence (34). Recent study suggested that M2-TAMs cocultured with PCa cells caused epithelial–mesenchymal transition and proliferation of tumor cells (35). Besides, the presence of M2-TAMs may be associated with the therapy resistance to immunotherapy for patients with mCRPC (15). M2-TAMs can secrete cytokines and pro-metastatic factors which prevent immune responses initiated by cytotoxic T cells, causing immunological silence (36). In addition, Guan reported that increased infiltration of M2-TAMs induced a drug resistance to docetaxel treatment for CRPC cells, and PLX3397 could restore the chemosensitivity by preventing the recruitment and M2-polarization of TAMs (14). However, the specific interactive mechanism between M2-TAMs and PCa is still incompletely understood.

Recent technological advances have allowed the development of various molecular prognostic indicators (37, 38). However, these tools are not necessarily universal, given that they are restricted to subsets of patients based on criteria such as hormone receptor, pathological types and nodal status (39, 40). It is urgent to explore better biomarkers or predictive tools for PCa. Based on WCGNA analysis, the modules and genes associated with the proportion of infiltrated M2-TAMs in PCa were explored in the present study. The results revealed that red module was the most relevant one. Functional annotation showed that biological processes and pathways were mainly associated with the immune-related processes, which was plausible and supported the result of WGCNA. Consequently, we explored the hub genes in red module. In the end, we identified ACSL1, DLGAP5, KIF23, and NCAPG as the hub genes with the degree of connectivity. Subsequent validation demonstrated that these four hub genes had a higher expression level in tumor tissues than that in normal tissues, and they were good prognosis biomarkers related to the proportion of infiltrated M2-TAMs for PCa.

High expression of ACSL1 was significantly related to poor outcome a variety of tumors. In hepatocellular carcinoma, ACSL1 promotes the fat metabolism of the tumor and the progression of the disease by catalyzing the ATP-dependent acylation of fatty acids into long-chain acyl CoAs (LCA-CoAs) (41). It was reported that decreased ACSL1 could suppress the protumorigenic inflammatory responses induced by granulocyte–macrophage colony-stimulating factor (GM-CSF) in breast cancer (42). In PCa, lipid metabolism regulated by ACSL1 rewires the PCa metabolome to support growth and resistance to endocrine therapies. Therapeutic strategies targeting lipid metabolism and androgen receptor are starting to emerge, providing new chances to re-sensitize tumors to endocrine therapies with lipid metabolic approaches (43). However, research regarding the effect of M2-TAMs on lipid metabolism of PCa is relatively limited. ACSL1 may be a potential target, when more studies confirm its values.

DLGAP5, which is mapped to chromosome 14q22.3, is a cell cycle regulator involved in carcinogenesis. DLGAP5 facilitates microtubules formation by working as a kinetochore protein (44). The expression of DLGAP5 is positively associated with the recurrence rate of PCa. Besides, it was observed that DLGAP5 was highly expressed in CRPC samples in many studies (45). Furthermore, Hewit’s study on PCa suggested that DLGAP5 played an important role in surviving microtubule assault from docetaxel and stabilizing spindle formation, in an androgen-regulated cell cycle system (46). Therefore, further studies on DLGAP5 were needed and were of great significance in clarifying the mechanism of how M2-TAMs cause chemoresistance and hormone resistance of PCa.

As a member of the kinesin superfamily, KIF23 was highly expressed in many kinds of tumors, such as gliomas, ovarian cancer, and gastric cancer (47, 48). In PCa, it has been proved that several members of KIF family play important roles in the process of metastasis, invasion and drug resistance (49, 50). In present study, KIF23 showed a high correlation with the proportion of infiltrated M2-TAMs, which meant that KIF23 may be a key gene in the interaction between M2-TAMs and PCa.

NCAPG is a subunit of the condensin complex, which is responsible for the stabilization and condensation of chromosomes during mitosis and meiosis (51). Studies suggested that NCAPG might be an essential oncogene of hepatocellular carcinoma and gliomas by inducing apoptosis (52). Similarly, Arai reported that abnormal expression of NCAPG promoted PCa cell aggressiveness (53). High expression level of NCAPG was observed in clinical samples of CRPC, and its expression was found to be important for PCa pathogenesis, as revealed by analysis of TCGA database. Our study suggested NCAPG might be a potential target, through which M2-TAMs affect the biological behavior of PCa cells.

There were limitations in our study that need to be recognized. First, the clinical information from the online database was not comprehensive, and the detailed information of some clinical treatment regimens also could not be obtained. Therefore, we could only evaluate the effect of these hub genes on overall survival to a certain extent, rather than that on other clinical endpoints. Second, we screened these hub genes by bioinformatics methods, where further experiments were required to verify and to determine the mechanisms underlying the process of malignant progression of PCa. Furthermore, the present study still did not identify a better mathematical model to combine all eligible hub genes together for predicting the survival. Therefore, future studies may aim for a better re-evaluation of the prognostic performance of the model for PCa.

## Conclusions

In conclusion, we identified four hub genes which were closely related to the proportion of infiltrated M2-TAMs in PCa. These findings contribute to understanding the underlying molecular mechanisms of how M2-TAMs affect PCa, and looking for the potential biomarkers and therapeutic targets for PCa patients. However, the molecular mechanism and function of these genes need to be confirmed in further experiments.

## Data Availability Statement

The original contributions presented in the study are included in the article/supplementary material. Further inquiries can be directed to the corresponding authors.

## Author Contributions

All authors listed have made a substantial, direct, and intellectual contribution to the work and approved it for publication.

## Funding

This study was supported by Startup Fund for scientific research, Fujian Medical University (Grant number: 2018QH1067), the Young and Middle-aged Talents Training Project of Fujian Provincial Health Commission (Grant number: 2019-ZQN-54), and Educational Research Programs for Young and Middle-aged Teachers of Education Department of Fujian Province (Grant number: JAT190204).

## Conflict of Interest

The authors declare that the research was conducted in the absence of any commercial or financial relationships that could be construed as a potential conflict of interest.
